# Energy-Efficient Wireless Weight Sensor for Remote Beehive Monitoring

**DOI:** 10.3390/s21186032

**Published:** 2021-09-09

**Authors:** Piotr Bratek, Piotr Dziurdzia

**Affiliations:** Faculty of Computer Science Electronics and Telecommunications, Institute of Electronics, AGH University of Science and Technology, Al. Mickiewicza 30, 30-059 Krakow, Poland; dziurdzi@agh.edu.pl

**Keywords:** apiculture, energy efficiency, IoT, maintenance of bee colonies, remote monitoring, wireless sensor nodes

## Abstract

This paper proposes a new approach to the construction of an autonomous weight sensor for electronic beehive scales, constituting a crucial part in equipment used in the modern beekeeping economy. The main goal of this work is to demonstrate a methodology at the preliminary design stage leading to saving scarce energy resources necessary for the remote operation of a wireless network of beehives. The main findings of the work, achieved results, and identified threats for beekeeping scales operating in the real environment are also shown. The results presented in the article are based on actual data collected and recorded from several dozen beekeeping scales operating in the natural environment over a period of several years.

## 1. Introduction

It is well known that bees, via their pollination of plants, have an immeasurable, positive influence on human life. Therefore, an increasing amount of scientists’ efforts are being directed not only toward a more detailed understanding of the physiology of bees but also toward providing them with appropriate living conditions and survival in the increasingly urbanized environment.

The extensive research covering bees’ evolution and physiology shows that the honeybee is uniquely predestined to integrative studies of behavior mechanisms [1]. The study conducted by Elekonich and Roberts provides a detailed discussion on the physiological and genetic mechanisms of honeybee behavior, which includes large-scale changes in hormonal activity, metabolism, flight ability, circadian rhythms, perception, and sensory processing. In [2], Abou-Shaara presents a review on foraging activity of bees, including the regulation of foraging tasks, factors impacting this behaviour, foraging preference, variations between subspecies, monitoring methods, and the possible methods for controlling this behavior.

More detailed research on the honeybee’s body properties and behavior can also be found in other scientific studies. For example, in [3], morphometric analyses of bee wings are presented and discussed; in [4], the body weight of honeybee drones and their changes in the development cycle are examined; and in [5], the influence of nutritional diet on the development of colonies, for example in stressful situations, is analyzed.

One of the technical sciences that supports the research of honeybee behaviors, and that is now confidently entering into the beekeeping community, is electronics, from simple bee counters to measuring appliances of environmental conditions inside and outside the hives, to advanced video systems as well as systems measuring the weight of hives and performing sound analysis in the hive. The application of apiary scales is established in the beekeeping industry, as they are used to determine the gains and losses of the hive mass and, in this way, indirectly indicate an increase or decrease in honey growth in a given bee colony.

A detailed study based on the results of bee scales is described in [6]. The analysis of daily fluctuations of the hive mass presented in this work was focused on the characterization of events occurring in a bee colony in specific periods of time. By using fragmentary regression modelling, predictable colony events were detected from the daily fluctuations in the colony mass.

A multi-sensor platform enabling real-time and long-term measurements of relevant parameters related to the conditions in the hives, such as the weight of the hive, sounds emitted by bees, temperature, humidity, and CO_2_ inside the hive, as well as outside weather conditions, is presented in [7]. The used electronic modules were described in detail; however, the issues regarding the operating range and distance, as well as the achieved working time of the device, were not considered, as the tests were carried out at a university campus. The work also investigated the spring and winter trends in hive mass changes, as well as the phenomenon known as swarming.

In [8] an initial implementation of a real-time IoT monitoring system is described; it was designed to measure the parameters of temperature, humidity, weight, and beehive smokiness (dedicated smoke sensor data were used). Another example of an apiary scale is described in [9]. The scale is based on one mass sensor with the HX711 amplifier and additionally included a sound module, gas sensor module MQ2, and a humidity and temperature sensor. A WiFi module and the MQTT protocol (Message Queuing Telemetry Transport) were used for transmission; unfortunately, the system required a high supply current of up to 2 A.

In [10] an approach with four mass sensors supporting the hive corners is presented. The sensors did not form a uniform structure, as they were separable and connected by cables to the main microcontroller; the measured data were transmitted via the GSM network. The authors concluded that the accuracy of just over 100 g was sufficient to monitor the nectar flow during the summer activity of bees. During the winter, they suggested increasing the accuracy in order to perform a more detailed analysis. Yet, another example in which four sensors were used working together in the system of one Wheatstone bridge can be found in [11]; the main scale module was based on the RasberryPi platform. In [12], an example of the implementation of a scale based on one load cell sensor is demonstrated. A weight sensor with a fairly large margin of measured mass was implemented, and a 24-bit ADC (Analog to Digital Converter) was used. A popular Atmega microcontroller was applied, and data were transmitted via the Zigbee interface. The paper also presents the power profile in specific operating states of the device.

To date, most of the considerations presented in the literature referring to beekeeping scales generally regard individual solutions and are focused on applying them to beekeeping farms only and demonstrating relatively simple and cheap implementations. For the last few years, many electronic beekeeping scales have appeared on the European market, offered both by professional electronic companies (e.g., [13,14,15,16]) and beekeepers and electronics hobbyists. Regarding the proposed method of measured data transmission, they were divided into two groups of scales: those dedicated to the home, and those dedicated to migratory apiaries. The first group is usually based on a short-range radio transmission, such as WiFi or Zigbee, or they directly indicate the measured weight of the hive on a display module; the second group uses globally available radio transmission technologies. In some countries, radio networks such as SigFox or LoRa are available, providing an undoubted advantage, namely the required relatively low energy demand joint with a long transmission range. However, the broadest available transmission coverage range is provided by GSM networks. Initially, these networks were only used to send a short text message to beekeepers via SMS, informing them about the state of the beehive; now, the GPRS transmission is more useful and provides much greater advantages of processing and analysis than before. Furthermore, uploading data to the cloud services is becoming a standard in professional approaches in the beehive remote monitoring field.

This paper proposes a new approach to the construction of an autonomous weight sensor used in the modern beekeeping economy. The main goal is to demonstrate a methodology at the preliminary design stage leading to saving scarce energy resources necessary for the remote operation of a wireless network of beehives. The main findings of the work are supported by actual data collected and recorded from several dozen beekeeping scales operating in the natural environment over a period of several years. The main novelty of the proposed solution is the structure of the scale based on four customized sensors, the original electronic hardware and innovative firmware allowing for a long working time with the use of low-cost batteries, as well as the original software for analysis in the cloud.

The article is organized as follows. In the next Section 2, we present identified preliminary requirements for a smart beehive scale based on the expectations of beekeepers. In Section 3, we show design steps leading to developing mechanical, electronic hardware as well as software parts of the beehive scale. The results of the tests performed in real environment followed by discussion can be found in Section 4 and Section 5. Additionally, the last Section 6 contains a summarizing conclusion.

## 2. Preliminary Requirements for a Smart Beehive Scale

In addition to measuring the weight of the hive (which was the main parameter suggested/raised by beekeepers during consultation), the smart beehive scales are also designed to deliver other parameters that are significant for the life of the bee colony, such as weather conditions outside the hive as well as temperature and/or humidity inside the hive. For example, external humidity informs whether there was rainfall; external temperature reflects the possible activity of bees outside a hive; internal temperature in the hive-depending on the location of the sensor, informs about the growth of the bee colony or the movement of bee swarms.

The mechanical assembly of the smart apiary scales should allow for easy installation. The construction should be light in weight and have a very stable structure, especially in the case of migratory apiaries, which are often translocated from place to place.

The beehive scales are expected to perform several measurements a day, with the ability to independently change the hours of measurements. Another significant requirement is a wireless operation range (coverage) that is as high as possible, even in remote places far away from inhabited areas. As the smart beehive scale works on scarce energy resources, such as typical replaceable batteries, the energy efficiency factors must be thoroughly taken into account to provide a life span that is as long as possible between battery replacements.

The identified requirements imposed some constraints and problems that had to be solved in interrelated electronic hardware, mechanical design, and cloud software. The following sections show in detail how these solution were identified.

## 3. Design of the Smart Beehive Scale

**Figure 1:**
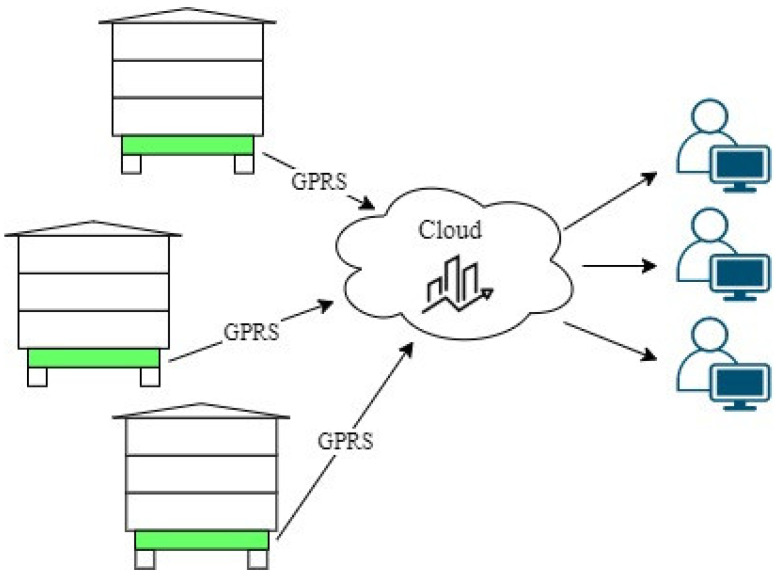
Idea of the smart beehive system for beekeepers.

The basic components of typical electronic scales are weight sensors, electronics for data processing and transmission control, and the transmission module [12]. The data are sent to a dedicated cloud that archives all the measurements and makes them available for further complex analysis. The cloud also stores configuration data for devices working in the network (Figure 1).

Strain gauges with a range of up to 100–150 kg are most commonly used in electronic scales as mass converters. In amateur versions of beehive scales, common weight sensors, the same as those found in bathroom scales, are used. Unfortunately, they suffer from very poor strength and are characterized by insufficient quality of material properties.

### 3.1. Mechanical Part of the Beehive Scale

**Figure 2:**
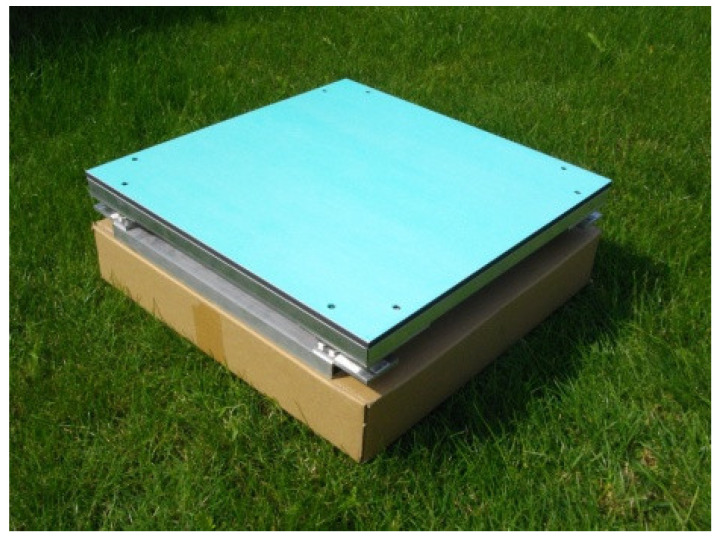
View of the designed electronic beehive scale placed on the ground [16].

Most of the professional scales use one sensor covering the entire range of the allowable weight of the hive. In this case, a base with stable balance is required, allowing the measured weight to be distributed fairly regularly. The authors propose the use of four strain gauges, significantly improving the stability and the balance of the structure as well as leading to better energy consumption properties. An image of one of the designed and realized beekeeping scales with implemented remote reading is presented in Figure 2 [16].

**Figure 3:**
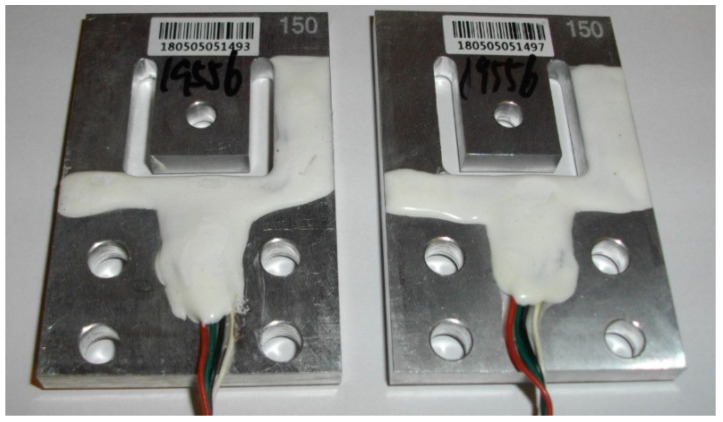
Strain sensors used in the smart beehive scale.

**Figure 4:**
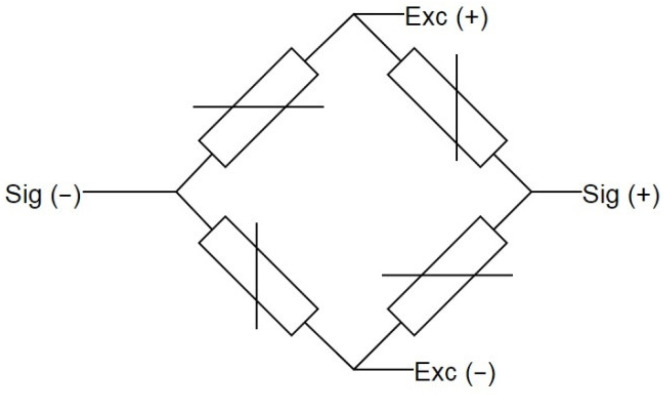
Load cell electrical connection in a Wheatstone bridge arrangement.

Four load cells—the strain sensors (Figure 3), each in a Wheatstone bridge arrangement (Figure 4)—were placed at the corners of the weighing platform. The weighing platform with dimensions of 45 cm × 45 cm was selected after analyzing the dimensions of the casing of the hives so as to make it suitable for the most typical wooden and polyurethane hives available on the market, as well as ensuring some margin.

**Figure 5:**
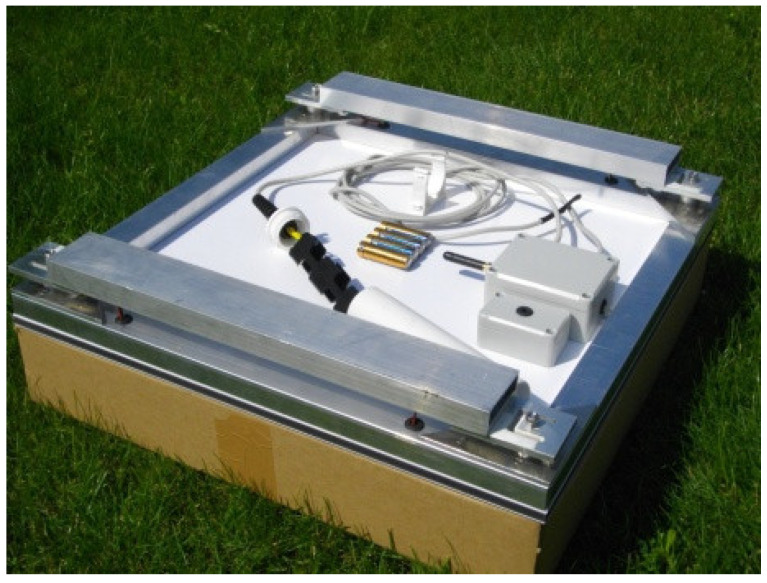
The bottom view of the beehive scale.

The mass sensors are connected to two beams/skids that form the basis of the entire device. Figure 5 shows the view of the designed weight platform from the bottom side. The proposed arrangement where there are as many as four support points in which the sensors are placed ensures much better stability than in the case of scales where there is only one sensor located in the center. The weighing pan frame as well as the skids are made of aluminum profiles, which ensures adequate strength and resistance to weather conditions, as well as relatively light total weight of the construction (only 3.5 kg).

### 3.2. The Electronic Part of the Beehive Scale

**Figure 6:**
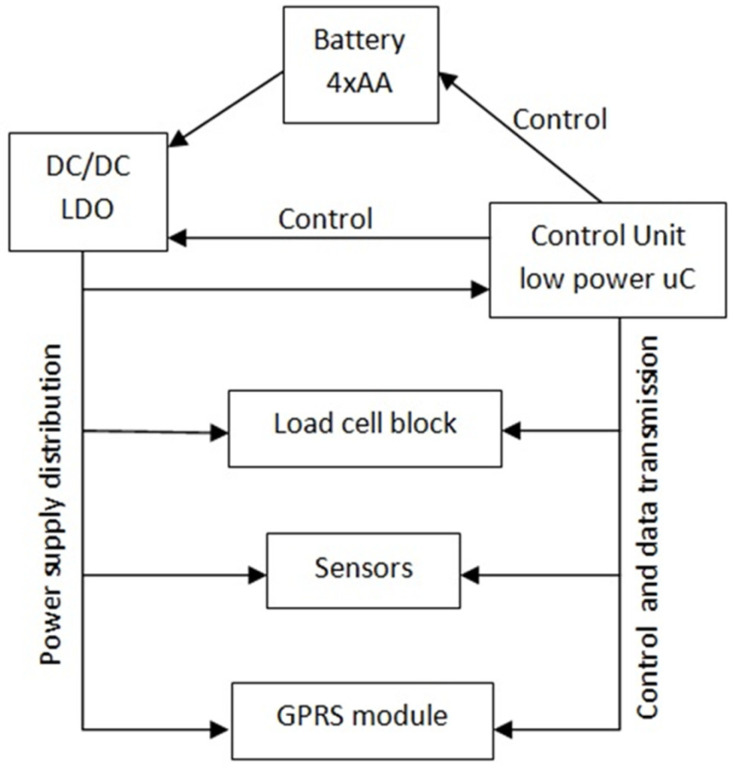
Block diagram of the electronic hardware of the beekeeping scale.

The general requirements defined at the beginning of the work for the electronics of the smart beehive scale allowed for the determination of the more detailed main electronic blocks (Figure 6). It was decided to use the GS standard for wireless communication, whereas for the power supply alkaline AA batteries were chosen, as one of the most popular, easily available, and inexpensive energy sources.

The main goals were to design and build an electronic system to control and manage the beehive scale using GPRS transmission and to maximize its operation time on a power supply consisting of only four AA alkaline energy sources. The devices available on the market usually run on gel 6 V and 12 V batteries, which unfortunately require cyclic charging. The proposed solution of power supply is easily available and inexpensive while significantly reducing the weight and dimensions of the scale system itself.

### 3.3. Selection of Components

**Figure 7:**
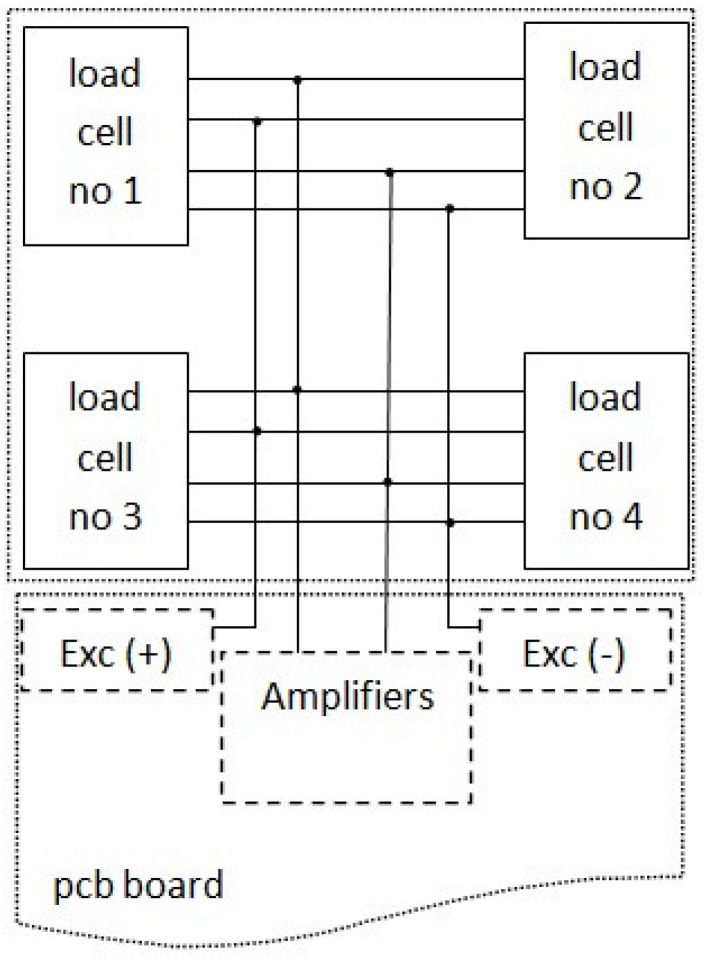
Connection diagram of strain sensors (planar beam load cell).

The main measuring instruments of the system are strain sensors—the load cells. Special sensors modified and customized by the manufacturer were selected so that they could sustain a very long-lasting mass load, exhibit nearly zero balance, and demonstrate a fast response time, as well as providing temperature compensation in the range of −10 °C to +40 °C; the resistance of individual sensor is approx. 350 Ohm. The voltage outputs from the load cells are connected to the amplifiers and next to the main board with a microcontroller to be subjected to analog-to-digital conversion at the end (Figure 7).

The core of the whole electronic system is a low-power microcontroller EFM32 from Silicon Labs [17], offering several energy-saving modes. The processor wakes up from a very low power sleep mode only for the time of taking measurements and controlling the GPRS transmission.

In order to provide appropriate voltages of the electronic modules and save the scarce energy resources, elements with a low leakage current were used, both during operation and in sleep mode. Additional digital temperature and humidity sensors were also selected for the necessary low-power operation. The sensors were only turned on for the short duration of the measurements and were then turned off.

**Figure 8:**
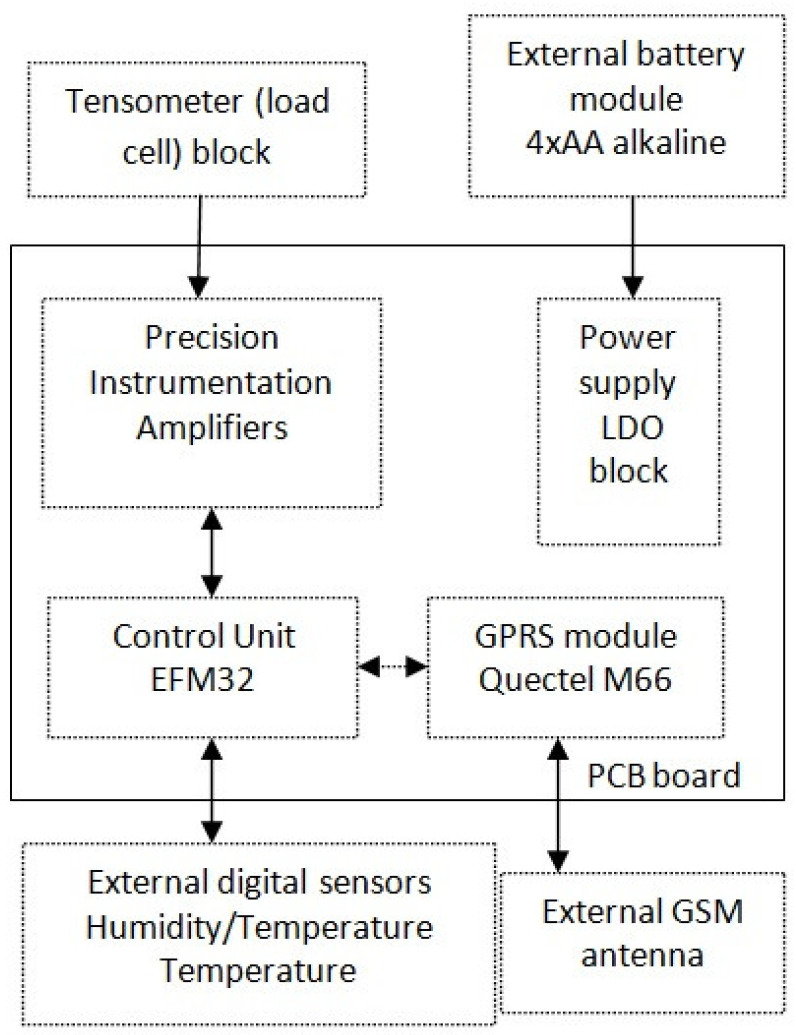
Block diagram of the board and connections of external elements.

**Figure 9:**
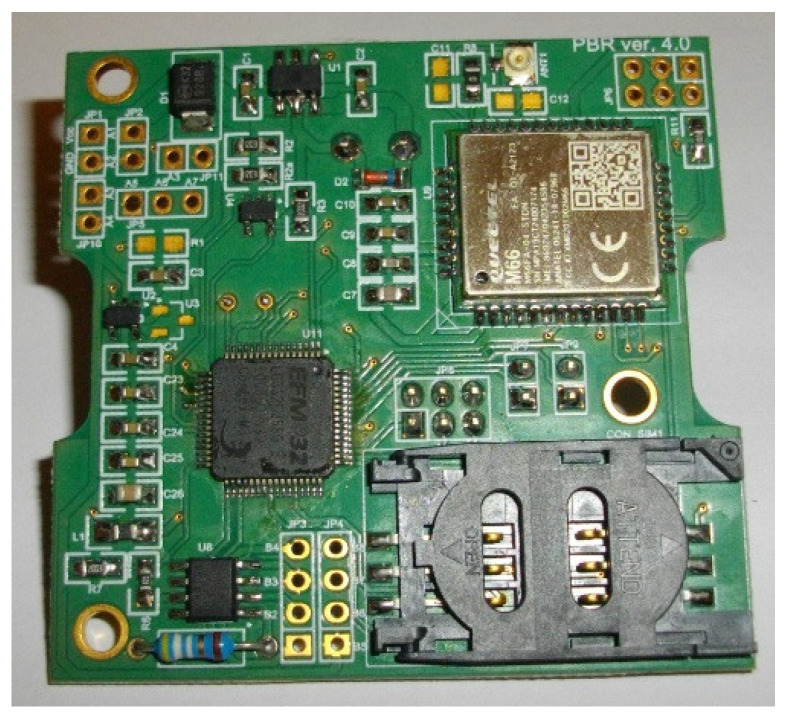
Assembled PCB of the beehive scale.

The data transmission is based on the Quectel M66 modem [18]. It performs wireless transmission deriving a very low electrical current peak (one of the lowest among the transceivers available on the market) at the so-called burst signals, which are typical for this type of transmission, reaching a value of approx. 1.6 A. In addition, the Quectel M66 makes it possible to set the transmission speed, which is correlated with the data transfer time. Instead of an extra RTC (Real Time Clock) system that is normally used for determining the periods of measurements and transmissions, the timings are managed in the system by the functionalities and internal timers of the microcontroller. The used microcontroller has internal 16-bit timers. It has also a 24-bit Real Time Counter and a 32-bit Backup Real Time Counter which are clocked by a 32.768 kHz crystal oscillator. Each time the beehive scale sends measurement data to the cloud, it also receives back the current time from the server, which allows for daily correction of any microcontroller time deviations resulting from the imperfections of internal timers and crystal oscillator. The MAX4194 [19] was used as a precision instrumentation amplifier to proportionally increase the small voltages from the load cells and adjust them to the inputs of the analog-to-digital converters. The selected amplifier is a micro-power, single-supply circuit that is dedicated to strain gauges with resistance in the range of 120–350 Ohm. The low dropout voltage regulator (LDO) block was based on circuits with a very low leakage current, in the range of 0.8–2.0 µA for lower supplied currents and 8–50 µA for higher current consumption [20]. Figure 8 shows a block diagram of the final electronic hardware part of the scale with connections to external modules, as well as data flow between the main parts of the system. The assembled PCB of the beehive scale is presented in Figure 9.

### 3.4. Software Part of the Beehive Scale

**Figure 10:**
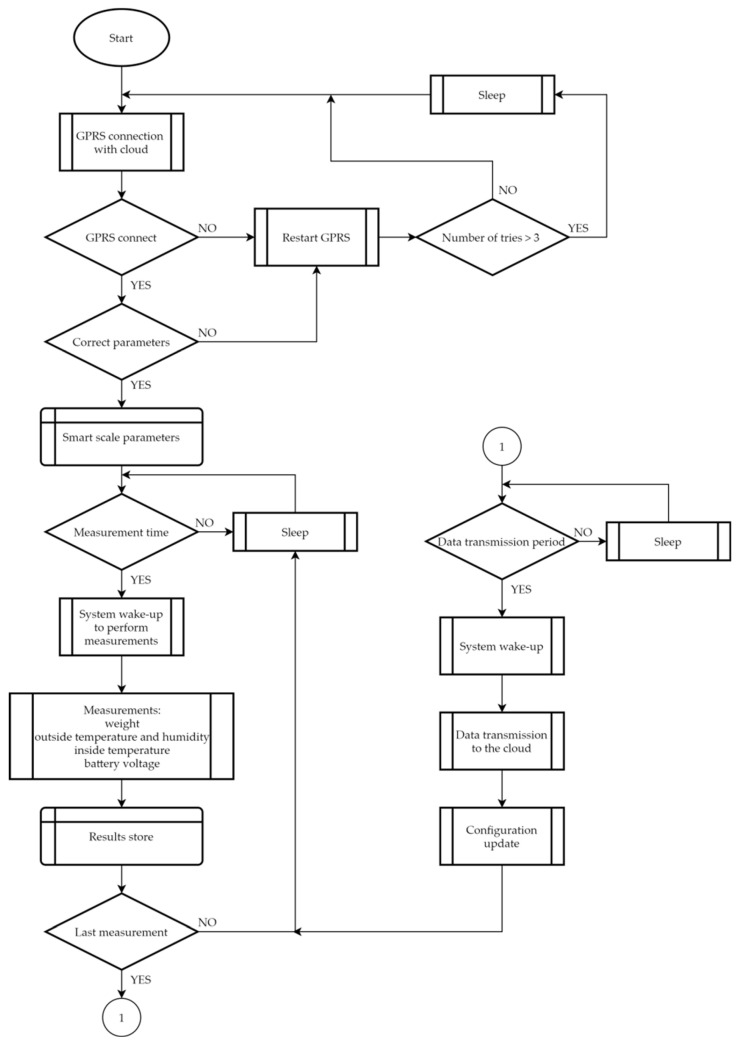
Software algorithm controlling the operation of the beehive scale.

The entire electronic system of the beehive scale is controlled by the microcontroller according to a proposed algorithm (Figure 10).

The algorithm of operation of the beehive scales was partly created on the basis of beekeepers’ opinions and expectations taken during numerous discussions and conversations. The foremost requirement concerned autonomous operation of the beehive scale for as long as possible. In addition, the following functionalities and requirements were expected: the ability to take several measurements a day; the range of the measured mass up to approx. 100 kg; the measurement resolution as low as possible (the weight measurement resolution is 0.1 kg); a large area of operation. The remote beehive scale performs three measurements of the defined/set parameters per day, and then, at the end, the data are sent via a GPRS channel to a dedicated cloud service.

After powering up the smart scale, the microprocessor system first makes connection to the cloud, where configuration data for each scale are stored; at the same time, it sends request information that requires updated configuration data. The configuration data include details regarding the hours of measurements to be taken, the time of transmission of the measured data to the cloud, and the current date and time. If the configuration data are not downloaded or there is a distortion during the transmission, the beehive scale goes into sleep mode after several attempts, because it is not identified whether the problem lies in the network signal quality or whether there is another reason. When the system starts and sets up the connection, the configuration data are sent to the microcontroller, which verifies their compliance with the required format of the transmitted data frame. When the received data frame is not compliant, then it indicates that possible distortion has occurred during transmission.

The aforementioned few attempts in the event of failure in downloading the configuration data represent a procedure that gives an opportunity to switch to another mobile operator. In the case of several unsuccessful attempts, the beehive scale enters into a deep sleep state and attempts to re-establish the connection after a few hours. If there is a problem with the base station, it is assumed that after some time it will be solved and the scale will be able to download the correct configuration. Only one SIM card is used in the modem of the beehive scale, which allows communication and data transmission in the networks of different operators (on the basis of international or domestic roaming). Switching between operators is possible by means of commands controlling the GPRS modem, namely the “AT + COPS” command, sent by the microcontroller to the modem; along with the appropriate parameters, it allows the operator to be selected.

After downloading the configuration data, the device saves it in the internal memory and goes into a deep sleep state. This state lasts until the measurements are to be taken, and then the microcontroller wakes up and performs the following measurements: mass, temperature, humidity, and battery voltage. Once the measurements are finished, the microcontroller saves the results in a flash memory and forces the system into sleep mode again until the next waking. The smart scale performs three measurements a day, and a few minutes after the last measurement, the measured and stored data are sent to the cloud. In order to maintain the continuity of data in the cloud (to prevent possible data loss and to make the system resistant to random problems with the coverage of the cellular network), the data sent to the cloud include always the results from the last three days.

After data transmission to the cloud, the beehive scale receives the current configuration data (for example, the user can change the hours of measurements). If the user has not introduced any changes, the scale updates the current date and time and then goes into sleep mode. If the user changes the hours of measurements, the microcontroller updates them in its memory and performs measurements on the next day in accordance with the new schedule. If the users note in the cloud resources that there has been no new data transfer for the last two or three days, they are advised to reset the scale or replace the battery. The current state of the battery is sent to the cloud and one can follow the changes in the battery voltage on the graph as well as monitor its capacity.

The average daily energy demand for the operation scenario presented above was only 17 mAh, assuming trouble-free GSM communication. The energy needed for GSM communication accounts for as much as 85% of the total demand. The designer has a very limited impact on the wireless quality and transmission distance/coverage. Therefore, it is important to efficiently manage the hardware system resources, especially the sleep periods, as well as optimizing measurements of the beehive parameters. Assuming the capacity of AA alkaline batteries at 2500 mAh, the estimated device operating time is 147 days.

### 3.5. Cloud Data Processing and Presentation

**Figure 11:**
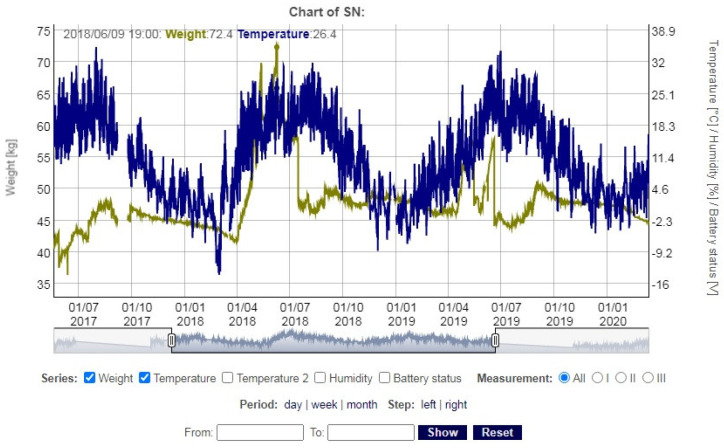
Sample data chart showing changes in mass and external temperature of the beehive scale.

A developed dedicated cloud environment for the proposed beekeeping scales allows for data recording, analysis and their presentation. The end-users, after logging into their account, can see the configuration data of their smart scales. They are able to set the hours of measurements, to name the scales individually, and to view the current battery status. All measured parameters, namely the hive weight, outdoor temperature and humidity, hive temperature and battery condition, including dates and times of measurements taken, are stored in database records. Users can view these records in the form of tables, as well as export them to a csv file and analyze them later on their own computer. Figure 11 shows sample linear data charts displayed in the web browser presenting the mass and the external temperature of the beehive scale.

**Figure 12:**
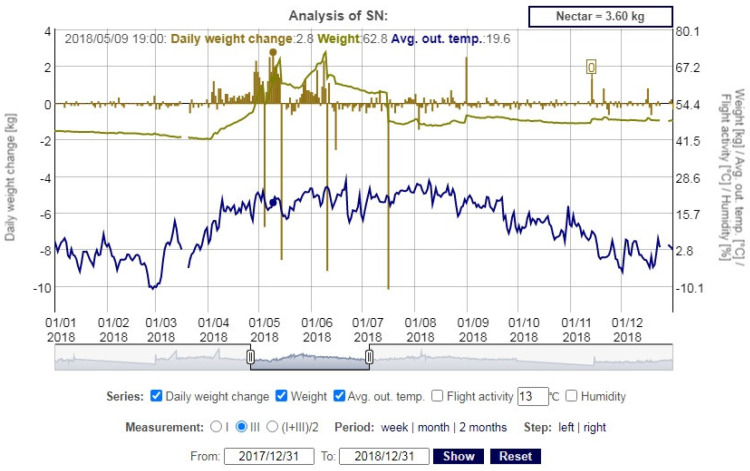
Sample chart of daily mass changes when the average outside temperature is taken into account.

Based on the transmitted data by beehive scales, users can observe the daily changes in the hive mass simultaneously with other measured parameters, which allows them to monitor the state of the bee colony in the current environmental conditions (Figure 12).

More advanced benchmarking comparative analysis is available for users whose devices operate over a longer period of time. They are able to compare individual months and years with each other, and to estimate the available nectar for bees and at the same time make corrections related to collecting honey and adding food.

## 4. Tests in Real Environment

The main problem observed during the period of several years of operation of the presented GSM electronic beehive scales with GPRS transmission in the real environment was the transmission distance and quality of the signal. Despite the almost full signal coverage of the country by the GSM network, there are still places with unfavorable conditions for setting-up reliable data links that would be suitable for monitoring beehive farms. In many locations, especially among arable fields or forests, the quality of the GSM signal is poor, which results in extended time necessary to choose an appropriate mobile operator and establish a connection for data transmission. All of this leads to higher power consumption and greater energy demand.

Another important issue, as predicted, was the problem of the weight of tare and its shift within operation time of the beehive scale, mainly due to the load cells remaining under the beehive load at all times. The results of the measured tare shifts for selected weights are presented in Table 1. The average tare level shift is at the level of 0.2 kg per 1 year, and it decreases in subsequent years, with an almost constant standard deviation of approx. 0.2 kg/year, which should be considered a satisfactory outcome. The maximum tare deviation occurred three years after taring, and it amounted to as much as 0.8 kg (approx. 0.27 kg annually). Therefore, it is recommended to check the zero offset operating point again after this period and the balance of tare if necessary.

Another important aspect that can significantly affect the measurement results is the influence of temperature on the readings of strain sensors. If the initial calibration is performed by the manufacturer, this problem was not visible. When the strain sensors are not preliminary calibrated, then the temperature drift has to be software compensated by means of an algorithmic linear function (resistive strain sensors exhibit linear dependence on temperature).

## 5. Results and Discussion

**Figure 13:**
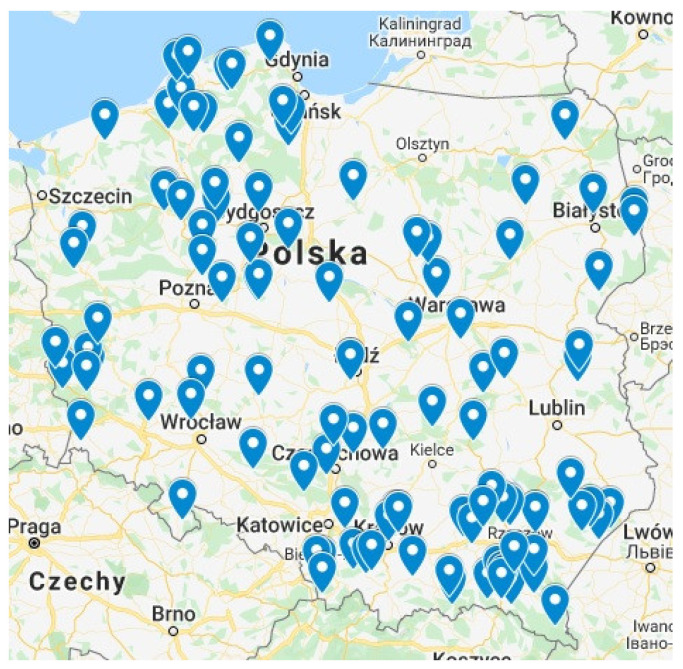
Map with locations of the deployed beehives equipped with designed smart scales.

The tests were carried out for selected 85 devices deployed in Poland and operating in the years 2017–2020. The cases where battery replacement resulted in losses in the continuity of measured and transmitted data were not taken into account during analysis. The locations of the deployed beehives equipped with the designed smart scales and participating in tests during the period of 2017–2020 and described in the previous section are shown in the map in Figure 13.

The data stored in the cloud make it possible to accurately determine when the battery replacement took place. The database saves each new beehive scale start-up in a separate table because the scale sends a request for configuration data. The logs stored in the cloud were thoroughly analyzed, and the differences between consecutive battery replacements were determined. In order to determine the battery voltage level, the measured data for the day preceding the battery replacement were used.

**Figure 14:**
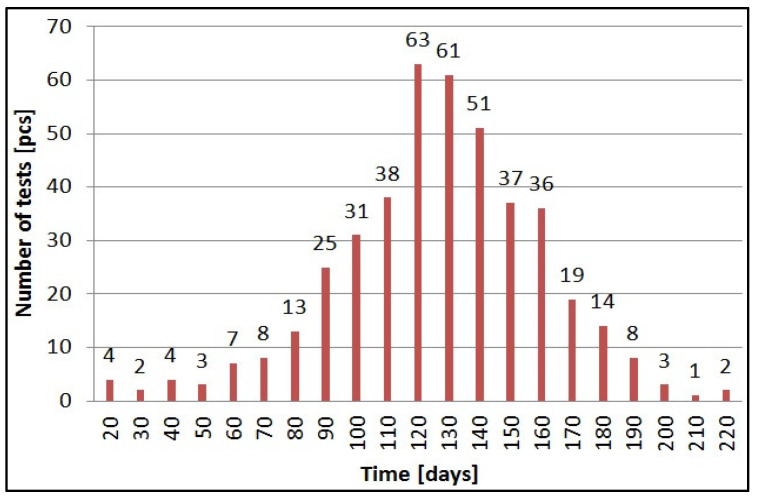
Actual lifetime of beehives deployed in different parts of Poland powered by a single packet of 4xAA batteries (number of tests 430 pcs).

The diagram below (Figure 14) shows the real operating time for the beehive scales powered by four AA alkaline batteries. The term “test” refers to lifetime (the full operation period) of a beehive scale, which is fed by (which runs on) one set of batteries. The presented data relate to 430 full periods of operation for 85 devices. The mean time duration of operation was 131 days with a standard deviation of 30 days.

**Figure 15:**
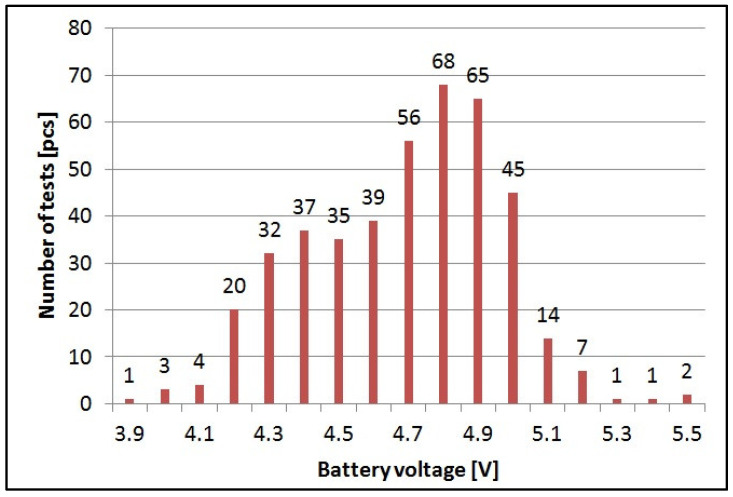
Battery voltage level at which correct data transmission was possible (average value 4.69 V, standard deviation 0.27 V).

Figure 15 shows the value of the battery voltage at which there was no data transmission from the beehive scale to the cloud. The value achieved during laboratory tests was at the level of 4.2 V, and it resulted from the electronic circuits constraints.

In real conditions, the absence of transmission was due to the lack of sufficient energy stored in the batteries to maintain the required supply voltage of the GPRS modem for the time of transmission to the cloud (too low voltage supplied to the modem causes its shutdown).

The operating time of the scales with 4 × AA power supply significantly varied depending on their location and the signal strength of the cellular network in a given area. In areas covered by a very low GSM signal, larger antennas had to be used, and, as such, the lifetime was only about 60 days. For areas with a strong signal, where the base stations were deployed close to the location of the beehive scales, the operating time was as long as 180–220 days. In most cases, the operating time on one set of alkaline batteries was on average 130 days.

## 6. Conclusions and Future Work

The presented concept of a low-energy beekeeping scale for monitoring beehive farms was practically implemented. Apart from 85 working scales that were subjected to analysis and the results presented in the article, several hundred more devices have been operating across Poland for several years. The assumed results were confirmed in practice; the beehives scales regularly performed the measurements of relevant data and sent them via the cloud environment to the beekeepers.

The mechanical construction based on four mass sensors with two supporting beams was the right choice, allowing high stability of the beehive system and offering the opportunity to place hives on row supports. The low-cost batteries powering the scales and their easy availability represent great advantages for beekeepers, especially when, in favorable conditions for GPRS transmission, the operating time on one set of batteries covers almost the entire period of main activity of bees in Poland, i.e., from April to September.

The opportunity to carry out analyses for given bee colonies is indispensable in the work of modern beekeeping farms and industry. The dedicated cloud service collects, archives, and makes data available to the user for analysis. In addition, it is possible to prepare dedicated charts and tables as well as communication with applications for mobile devices.

In addition to the rapid development of cellular techniques, a gradual increase in the popularity of LPWA techniques is also expected (e.g., NB-IoT and Cat.M), which guarantees a very large area of reliable operations of beekeeping scales (thanks to the global coverage offered by mobile network operators), with a substantial decrease in energy demand due to significant limitations of current peaks occurring during data transmission.

Future work will focus on the further optimization of the energy consumption of the beehive scales, providing alternative sources of energy (energy harvesting) while maintaining their global range of operation.

## Figures and Tables

**Table 1:** The deviations/offset/tare level in kg.

Time from Taring	1Y	2Y	3Y	4Y
Probes	22	17	10	2
Average tare deviation	0.227	0.288	0.33	1.15
Standard deviation of average tare	0.1956	0.2027	0.2627	0.212
Average tare deviation per year	0.227	0.144	0.11	0.038
Maximum tare deviation	0.6	0.6	0.8	0.3
Maximum tare deviation per year	0.6	0.3	0.27	0.075

## Data Availability

Not applicable.
